# Determination of Volatile Organic Compounds (VOCs) from Wrapping Films and Wrapped PDO Italian Cheeses by Using HS-SPME and GC/MS

**DOI:** 10.3390/molecules19078707

**Published:** 2014-06-25

**Authors:** Sara Panseri, Luca Maria Chiesa, Alfonso Zecconi, Gabriella Soncini, Ivano De Noni

**Affiliations:** 1 Department of Veterinary Science and Public Health, Università degli Studi di Milano, Via Celoria 10, Milan 20133, Italy; E-Mails: sara.panseri@unimi.it (S.P.); alfonso.zecconi@unimi.it (A.Z.); 2 Department of Veterinary Science and Technologies for Food Safety, Università degli Studi di Milano, Via Celoria 10, Milan 20133, Italy; E-Mail: gabriella.soncini@unimi.it; 3 Department of Food, Environmental and Nutritional Sciences, Università degli Studi di Milano, via G. Celoria 2, Milan 20133, Italy; E-Mail: ivano.denoni@unimi.it

**Keywords:** PDO cheese, cling -films, polyvinylchloride (PVC), polyethylene (PE), food safety, VOCs, SPME, GC-MS

## Abstract

Nowadays food wrapping assures attractive presentation and simplifies self-service shopping. Polyvinylchloride (PVC)- and polyethylene (PE)-based cling-films are widely used worldwide for wrapping cheeses. For this purpose, films used in retail possess suitable technical properties such as clinginess and unrolling capacity, that are achieved by using specific plasticizers during their manufacturing process. In the present study, the main VOCs of three cling-films (either PVC-based or PE-based) for retail use were characterized by means of Solid-Phase Micro-Extraction and GC/MS. In addition, the effects of cling film type and contact time on the migration of VOCs from the films to four different PDO Italian cheeses during cold storage under light or dark were also investigated. Among the VOCs isolated from cling-films, PVC released 2-ethylhexanol and triacetin. These compounds can likely be considered as a “non-intentionally added substance”. These same compounds were also detected in cheeses wrapped in PVC films with the highest concentration found after 20 days storage. The PE cling-film was shown to possess a simpler VOC profile, lacking some molecules peculiar to PVC films. The same conclusions can be drawn for cheeses wrapped in the PE cling-film. Other VOCs found in wrapped cheeses were likely to have been released either by direct transfer from the materials used for the manufacture of cling-films or from contamination of the films. Overall, HS-SPME is shown to be a rapid and solvent free technique to screen the VOCs profile of cling-films, and to detect VOCs migration from cling-films to cheese under real retail storage conditions.

## 1. Introduction

Polyvinylchloride (PVC) and polyethylene (PE) cling-films are widely used worldwide for cheese wrapping [1]. The major end-users are supermarkets where cheeses are sold either wrapped in film or overwrapped on a polystyrene tray. Nowadays, food wrapping provides an attractive presentation and facilitates self-service shopping. Films utilized for wrapping food, especially in retail, must possess tailored properties such as clinginess and unrolling capacities. For these reasons, manufacturing of wrapping film requires use of additives and ingredients capable of enhancing the technical properties of the co-polymer [2]. Hence, hundreds of plasticizers have been used to impart flexibility to plastics such as PVC. On the contrary, no plasticizers are needed for PE film as its flexibility is high enough even if other properties (in particular ‘clinginess’) are poorer than those of PVC films [2,3,4]. It has been apparent for several years that the use of cling-films in contact with fatty foods such as cheese may lead to migration of additives from the film to the food [5]. Hence, European legislation defines positive lists including compounds usable as monomers or additives for food contact plastics [6]. This legislation regulates migration from food-contact materials into foods by establishing an overall migration limit applicable to the total of the migrating compounds. At the same time, use of compounds which are necessary for the production of plastics but which may be harmful to human health is restricted by defining specific migration limits (SMLs). In retail and at home, wrapped cheese is commonly stored in small pieces with a high ratio of contact-surface area/volume. Moreover, the film generally overlaps the cheese surface which is actually covered by two or more layers [7]. For these reasons, migration under real retail/domestic storage conditions could increase overall migration. Volatile organic compounds (VOCs) are compounds characterised by a very low boiling point. Sources of some VOCs in food include packaging from which they can be transferred [8,9,10]. To date, little has been published on VOCs potentially released by cling-films used in retail or at home [11]. Moreover, scant information is available about migration of VOCs to cheese under conventional storage conditions in retail. The aim of this research was to characterize the main VOCs from three cling-films for retail uses. In addition, the effects of retail film type and contact time on the migration of VOCs from three films to four different PDO Italian cheeses during cold storage in a cold cabinet are also presented. Headspace Solid-Phase Micro-Extraction (HS-SPME) in combination with gas chromatography-mass spectrometry (GC-MS) was used for the identification of the VOCs in this study. HS-SPME is a rapid and sensitive method of sampling and preconcentration of volatile and semi-volatile compounds, which is being routinely used in combination with GC/MS. [11,12,13]. It is an inexpensive, solvent-free, and reliable technique with excellent sensitivity and good selectivity [14]. Considering the lack of information available in the literature regarding migration from packaging materials into real fatty matrices like cheese the aim of the present research was to use SPME to study the VOCs that are likely to migrate from cling-films to wrapped cheese.

## 2. Results and Discussion

### 2.1. Selection of SPME Fiber Coating and Extraction Time

**Figure 1:**
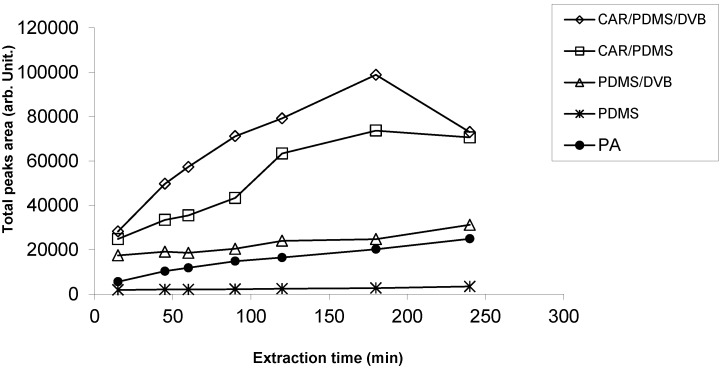
Total VOCs peak area (arbitrary unit; area/10^5^) for CAR/PDMS/DVB, CAR/PDMS, PDMS/DVB, PDMS and PA fibers at different extraction times (15, 45, 60, 90, 120, 180, 240 min).

The total VOCs area (arbitrary units) obtained at different extraction times in combination with different SPME fibers is presented in Figure 1. The best absorption was obtained at 180 min. In particular, the two fibers DVB/CAR/PDMS and CAR/PDMS displayed the highest sorption capacity compared to PDMS/DVB and PA fibers. An increase also was observed in the VOC adsorption increasing extraction time until 180 min. Finally the DVB/CAR/PDMS fiber was chosen also because it was very sensitive to small molecules like xylenes, ethylbenzene, styrene present in different cheeses. Indeed, the CAR/PDMS/DVB fiber has been reported to possess greater affinity for aromatic and aliphatic hydrocarbons and alcohols than CAR/PDMS and PDMS [15,16,17,18].

Once optimized the SPME extraction conditions, the analytical parameters of the method (linearity, limit of detection (LOD), limit of quantification (LOQ), precision and accuracy) were determined to validate the SPME-GC/MS procedure for determination of selected VOCs migrated from cling-films to cheeses.

### 2.2. Linearity, Precision, LOD, LOQ and Accuracy

The proposed method showed a good linearity with determination coefficients (r^2^) higher than 0.9970 for all investigated compounds, confirming the suitability of CAR/PDMS/DVB fiber to monitor VOCs in wrapped cheese samples with excellent linearity. Accuracy was estimated by recovery assay. The recoveries calculated using the matrix-matched calibration curves were in the range of 82% and 95% for all selected VOCs. The detection and quantification limits of VOCs (LOD and LOQ) as well as all validation results were presented in Table S1 (Supplementary Materials). Precision of the method expressed as the relative standard deviations (R.D.S.) obtained from the determination of six SPME cheese extracts (each prepared separately from a representative pool of cheese samples) showed values less than 5.5% for all selected compounds. These results confirm the efficacy of SPME method for the determination of VOCs compounds migrated from cling-films to PDO cheeses.

### 2.3. VOCs of Cling-Films for Retail Use

**Figure 2:**
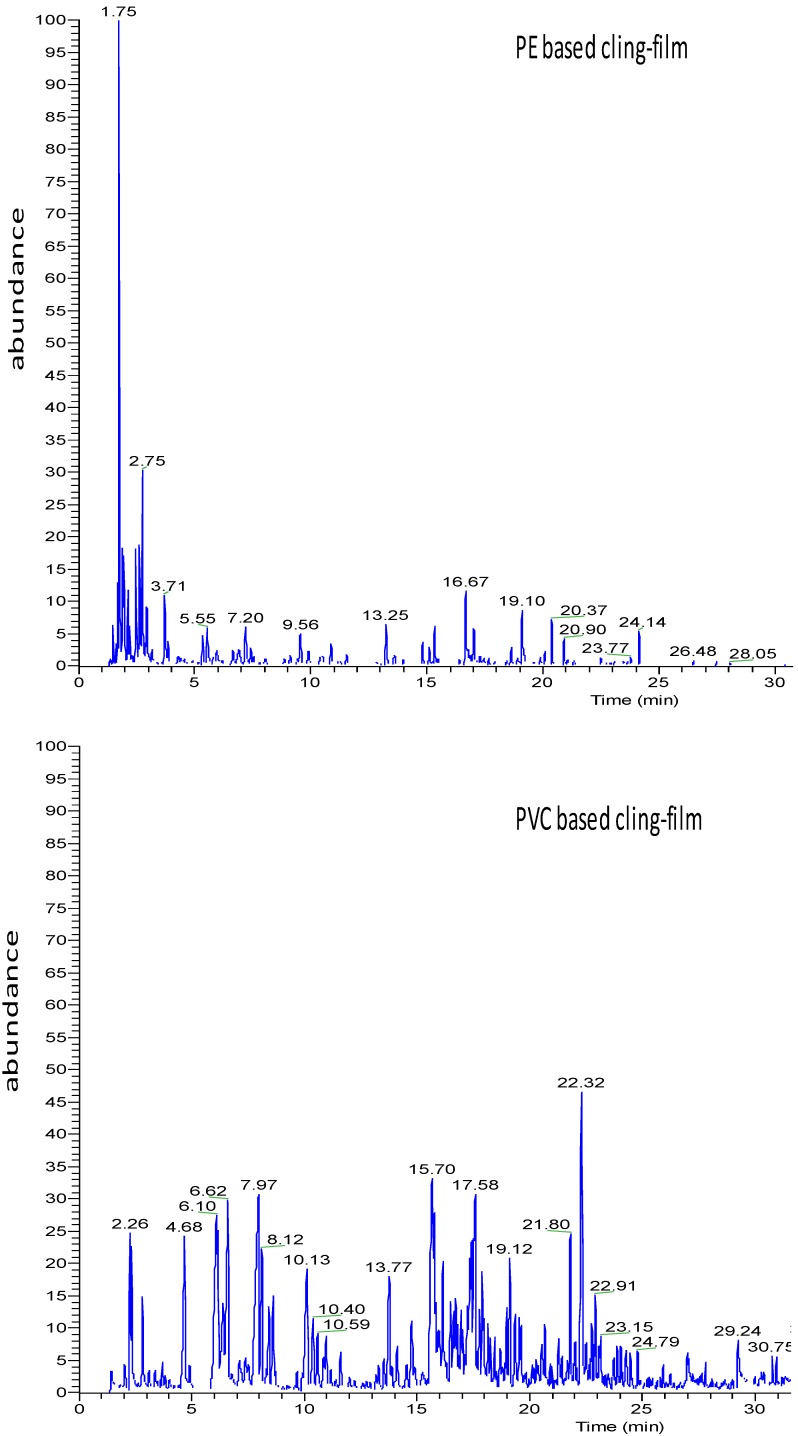
Chromatograms (total ion current-TIC) of PE based and PVC based cling-films.

The different VOC profiles characterized PE-based cling-film (PE200) and PVC-based cling-films (812 and 818) are presented in Table 1 and Figure 2.

**Table 1:** VOC profile by HS-SPME-GC/MS of cling-films for retail use.

Rt ^a^	Volatile Compounds ^b^	Cling-Films for Retail Use ^c^					
		812 PVC Based		818 PVC Based		PE 200 PE Based	
		mean	s.d.	mean	s.d.	mean	s.d.
		(*n* = 3)	(±)	(*n* = 3)	(±)	(*n* = 3)	(±)
	*Hydrocarbons*						
1.63	Heptane	4.13	0.09	nd	-	nd	-
1.71	Cyclohexane	nd	-	nd	-	34.89	0.79
1.86	Methylcyclohexane	0.80	0.02	nd	-	20.38	0.46
2.28	1-Chlorobutane	29.06	0.66	8.14	0.18	nd	-
4.16	2,2,4,6,6-Pentamethylheptane	nd	-	7.52	0.17	nd	-
5.17	Decane	nd	-	0.00	0.00	28.76	0.65
6.1	Toluene	nd	-	3.79	0.09	5.56	0.13
10.35	Ethylbenzene	3.33	0.08	3.51	0.08	21.33	0.48
10.95	*p*-Xylene	1.40	0.03	2.21	0.05	7.34	0.17
10.99	*m*-Xylene	10.80	0.24	24.05	0.54	1.47	0.03
11.05	*Oo*-Xylene	4.25	0.10	13.20	0.30	0.78	0.02
16.18	Styrene	4.41	0.10	14.29	0.32	1.01	0.02
total		58.18		76.72		121.51	
	*Aldehydes*						
2.61	Butanal	2.09	0.05	1.60	0.04	nd	-
4.33	3-Methylbutanal	2.75	0.06	1.74	0.04	nd	-
8.25	Hexanal	22.95	0.52	12.40	0.28	4.66	0.11
13.38	Heptanal	11.32	0.26	8.15	0.18	3.82	0.09
17.44	Octanal	4.22	0.10	3.67	0.08	11.61	0.26
20.47	Nonanal	66.48	1.50	46.29	1.05	51.77	1.17
21.22	2-octenal	2.63	0.06	1.29	0.03	nd	-
23.04	Decanal	13.45	0.30	nd	-	nd	-
total		125.88		75.15		71.86	
	*Ketones*						
2.1	2-Propanone	1.32	0.03	nd	-	nd	-
11.84	3-Heptanone	8.32	0.19	8.60	0.19	5.44	0.12
total		9.64		8.60		5.44	
	*Esters*						
2.77	Acetic acid ethyl ester	nd	-	5.75	0.13	0.00	0.00
2.84	Acetic acid vinyl ester	2.56	0.06	7.65	0.17	12.82	0.29
5.63	Formic acid butyl ester	0.93	0.02	0.39	0.01	nd	-
8.01	Acetic acid butyl ester	8.31	0.19	5.41	0.12	nd	-
20.38	Acetic acid 2-ethylhexyl ester	nd	-	9.79	0.22	nd	-
33.01	Triacetin	9445.71	213.60	7808.72	176.58	nd	-
34.31	Diacetin	26.92	0.61	28.41	0.64	nd	-
34.69	Monoacetin	11.60	0.26	11.63	0.26	15.81	0.36
total		9496.03		7877.75		28.63	
	*Alcohols*						
11.65	1-Butanol	15.69	0.35	13.01	0.29	3.18	0.07
19.5	2-Ethylhexanol	0.95	0.02	1.22	0.03	nd	-
total		16.64		14.23		3.18	
	*Terpenes*						
5.49	α-Pinene	1.50	0.03	0.85	0.02	28.76	0.65
12.73	β-Myrcene	19.62	0.44	19.07	0.43	17.51	0.40
13.85	D-Limonene	222.07	11.81	255.53	10.30	225.21	9.62
16.7	*p*-Cymene	1.62	0.04	1.19	0.03	nd	-
22.57	Citronellol	2.13	0.05	57.60	1.30	41.86	0.95
24.2	Linalool	10.71	0.24	18.46	0.42	16.30	0.37
total		257.65		352.71		329.64	
	*Miscellaneous*						
21.64	Acetic acid	88.19	1.99	112.00	2.53	19.59	0.44
35.45	2,4-di-*tert*-Butylphenol	nd	-	nd	-	10.25	0.23
total		88.19		112.00		29.84	

^a^ Retention time; ^b^ Volatile compounds: mass spectra tentatively identified using NIST 05 and Wiley 275 libraries; ^c^ Amount of volatile compounds expressed as µg IS equivalents g^−1^ of plastic film; samples (*n* = 3); s.d. = standard deviation (*n* = 3); nd = not detected.

**Figure 3:**
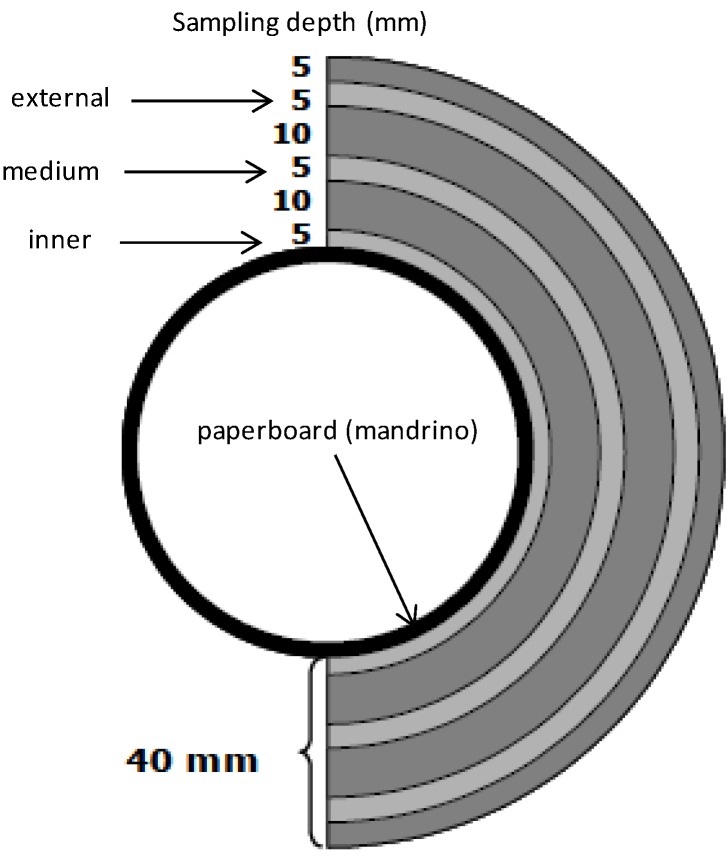
Scheme of sampling of cling-film at different depths of the roll.

**Figure 4:**
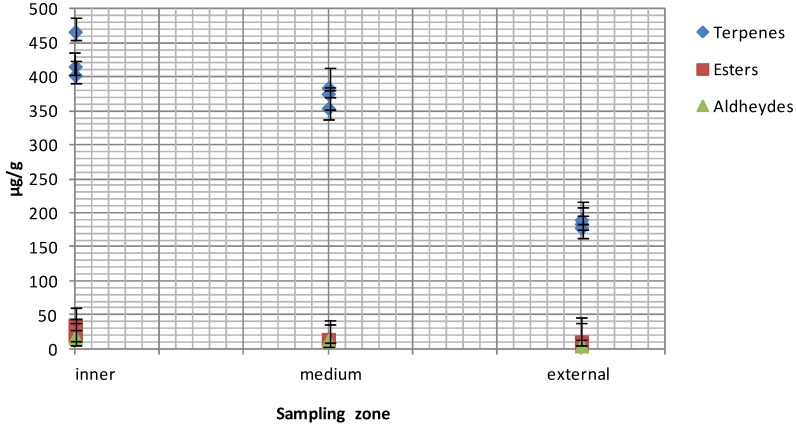
Terpenes, esters and aldehydes content in different sections of rolls (µg g^−1^).

The extensible films used to wrap foods in both domestic and retail contexts are usually made from PVC, polyvinylidene chloride (PVDC) or PE [19]. The desired mechanical characteristics (ﬂexibility, extensibility) are obtained by using various additives like plasticizers, mainly represented by adipates, citrates, and phthalates [20,21]. Authorized compounds are specifically listed in Annex I of Regulation 10/2011/CE) [22] even if compounds not included may still be present as a “non-intentionally added substance” (NIAS). These substances are decomposition products or reaction intermediates of the authorized starting additives [23,24,25]. On these bases, the three cling-films were initially assessed for global migration according to Regulation 10/2011. All the films studied fulfilled this Regulation (Table S2, Supplementary Material). HS-SPME and GC/MS were then applied to ascertain the VOCs’ released by cling-films. Presence of some VOCs like 2-ethylhexanol (2-EH, CAS 104-76-7) may result both from residue in cling-films and degradation of plasticizers. Indeed, 2-EH is a starting material in the production of low-volatility esters, such as the plasticizer di-(2-ethylhexyl) phthalate (DEHP) [22]. Moreover, 2-EH can be used in the production of 2-ethylhexanoic acid (2-EHA) and its calcium and zinc salts are used as heat-stabilizers during manufacturing of PVC films [26]. To date, the use of 2-EHA is not forbidden for manufacturing of food-contact material despite some concerns raised about its potential genotoxicity [27]. Among isolated VOCs, PE 200 sample did not release 2-EA and the triacetate ester of glycerol (triacetin, CAS 102-76-1). To our knowledge, no data are available in the literature concerning the presence of 2-EHA in cheese. An ester of adipic acid and 2-EH (di-(2-ethylhexyl) adipate, DEHA) is used as a plasticizer during manufacturing of PVC-based films. This compound could represent a degradation product arising from PVC manufacturing and, therefore, it can be considered as a NIAS [25]. Tri-, di- and monoacetin were present only in PVC-based films (812 and 818) as previously found in films for in-home use (data not shown). Triacetin was used for manufacturing food-contact plastics until 2009, as stated by Italian Regulation (Direttiva del Ministero Salute 23/4/2009) [28]. Triacetin possesses antifungal properties and it is used as a food additive (E 1518) for the solubilisation of humectant flavours in perfumery and cosmetics. To date, data concerning release of triacetin from food-contact plastics are not available even if, based on the available literature, this substance does not represent a hazard to human health [29,30]. Indeed, triacetin was generally recognized as safe (GRAS) by the Food and Drug Administration (FDA) [30]. For these reasons, its exclusion from the positive lists provided by European directives and regulations cannot be easily explained. Probably, due to its antifungal properties, triacetin could be regarded as an “active substance” and, therefore, it should meet the requirements provided by EU Reg. 450/2009 for active and intelligent packaging [31,32]. The PE 200 film released 2,4-bis(1,1-dimethylethyl)phenol (2,4-DTBP, CAS 96-76-4), an antioxidant added to plastics to inhibit or slow oxidative processes, while being itself oxidized. This antioxidant is also used as an intermediate for the preparation of UV stabilizers [33]. This compound is not included in any of the positive lists provided by European regulations and directives [34]. Different terpenes as well as aromatic hydrocarbons were present among VOCs extracted from cling-films (Table 1). In particular, the presence of limonene, linalool and citronellol is likely to be associated either with the storage conditions of films or migration from food packaging materials manufactured from waste or recycled food packaging like the internal (mandrino) or external cardboard part of a film roll (supplementary material) [35]. It is worth noting that no European regulations or directives exist for the use of recycled materials for food packaging. To date, mandrino and boxes must be manufactured using only virgin cellulose [35]. Only article 2 of directive 89/109/EEC requires that the consumer’s health must not be endangered by food-contact material. The aromatic hydrocarbons styrene (ST, CAS 100-42-5) and xylenes (CAS *p*-XY, CAS 106-42-3; *o*-XY, CAS 65-47-6; *m*-XY, CAS 108-38-3) were also found in cling films. Several contaminants (aldehydes, alkanes, hydrocarbons, and ketones and trace elements) have also been detected arising from paper board materials made of recycled fibres [35,36]. Although not intended, these compounds may be found in the final recycled material, it is very difficult to remove them efficiently, and no specific requirements and limitations are provided by the European Union [35]. On these bases, the cling-films studied were sampled at different depths of the roll (Figure 3). Amounts of some VOC classes observed in the different sections are shown in Figure 4. In particular, the internal cling-film portion (close to the mandrino) was richer in aldehydes, esters and terpenes probably as a consequence of migration phenomena from the board. To avoid interferences arising from the middle and internal part of the film roll, only the external part of the rolls was used for studying migration phenomena from the cling-films to wrapped cheese (Figure 3).

### 2.4. VOCs Isolated from Cheeses Wrapped in Cling-Films for Retail Use

The migration of VOCs depends on the characteristics of the polymer and of the food [37,38]. From this point of view, cheese can be considered a complex food matrix which undergoes several (bio)chemical and rheological changes during storage. For this reason, samples of cheese were chosen so as to account for different manufacturing procedures which could lead to different features of the derived matured cheeses. Indeed, as expected, several and different VOCs (aldehydes, free fatty acids and some ketones) found in cheeses derived from biochemical processes involved in their ripening. For this reason, only VOCs never reported as naturally present in cheese and clearly attributable to migration phenomena from cling-films were considered. Among them, only VOCs raising particular health or legal issues are discussed below.

**Figure 5:**
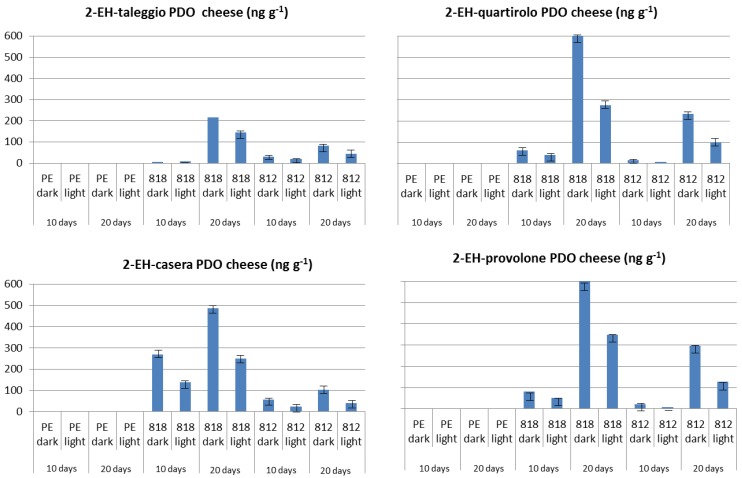
Levels of 2-ethylhexanol (2-EH) in PDO cheeses wrapped in different cling-films and stored refrigerated for 10 and 20 days in the dark or under fluorescent light (data expressed as ng·g^−1^ of cheese).

The effect of storage time and film type as well as that of light on VOC levels in wrapped cheeses was not always clearly inferable. Nonetheless, as expected, only cheese samples wrapped in PVC-based films contained 2-EH with the highest levels of being present in cheese samples stored for 20 days and kept in the dark (Figure 5). The release of this VOC during storage appeared quite linear and its content in wrapped cheeses almost doubled from 10 to 20 days dark storage.

2-Ethylhexanol can easily migrate from film to food and directive 2002/72/CE provides a SML equal to 30 mg·kg^−1^ food [31]. To verify this limit, cheese samples wrapped in PVC-based films and stored (10-d) in the dark, as well as the sample of Quartirolo stored 20 days under light, were submitted to quantitative determination of 2-EH following the method provided by the Community Reference Laboratory for Food Contact Materials (http://ihcp.jrc.ec.europa.eu/) [31]. All the cheese samples respected the SML stated by directive 2002/72/CE.

**Figure 6:**
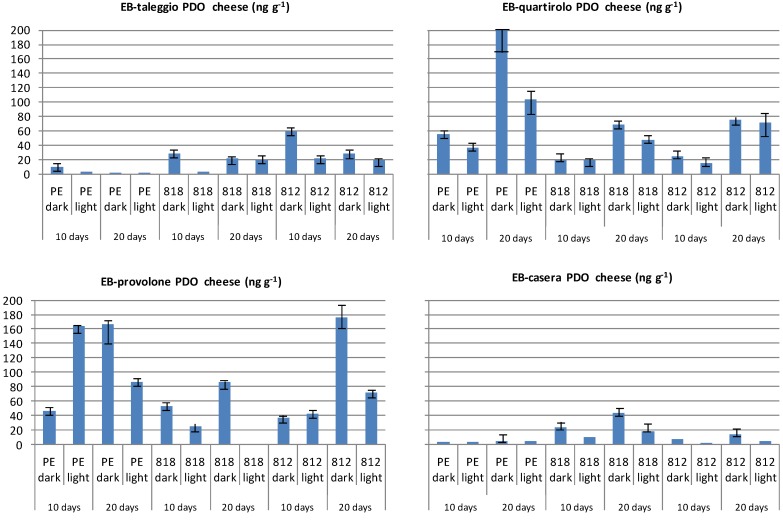
Levels of ethylbenzene (EB) in PDO cheeses wrapped in different cling-films and stored refrigerated for 10 and 20 days in the dark or under fluorescent light (data expressed as ng·g^−1^ of cheese).

Only limited data are available for ethylbenzene (EB, CAS 100-41-4) contents in non-packaged foods and, for instance, EB was detected in milk [39,40]. Levels of EB in the studied wrapped cheeses are reported in Figure 6. Slight evidence of the effects of storage time and lighting can be argued for the migration of EB, which was not present in unwrapped cheeses.

EB occurs in nature as a fraction of petroleum and is a synthetic chemical used as a precursor for styrene and polystyrene [39,40]. It should be mentioned that EB is also a common pollutant of air where it can easily be found as a consequence of industrial emissions [40,41]. On this basis, its presence in the studied cheese samples can be attributed to both migration and environmental pollution. However, levels of EB in the air surrounding the cabinet display were not monitored and, therefore, some of the EB found in cheeses could derive indirectly from air-to-film migration. Overall, the highest levels of EB were recorded in wrapped Provolone followed by Quartirolo (Figure 6).

Levels of EB in cheeses wrapped in PE-based film (PE 200) augmented with storage time, with darkness a promoting factor in raising EB levels in cheeses. The enhancing effect of darkness and time was evident also for Provolone and Casera cheeses wrapped in PVC films (812 and 818). On the contrary, a positive role of darkness only can be inferred for Taleggio and Quartirolo samples, since the prolongation of storage time decreased the EB levels in cheeses stored in absence of light.

**Figure 7:**
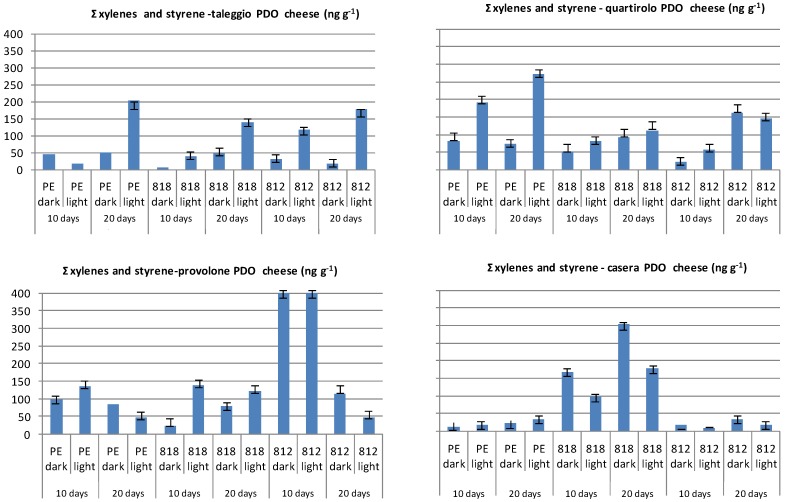
Levels of xylenes (Σ of *p*-*m*-*o* xylene) and styrene in PDO cheeses wrapped in different films and stored refrigerated for 10 and 20 days in the dark or under fluorescent light (data expressed as ng·g^−1^ of cheese).

The main source of oral exposure to styrene was reported to derive from migration from polymer packaging materials [35]. Low concentrations of styrene (0.1 μg·kg^−1^) were found in milk and cheese whereas higher concentrations (up to 5 mg·kg^−1^) were detected in some moldy cheeses since this VOC can be produced by fungi [40]. Higher concentrations can be observed as a result of styrene migration from polystyrene or styrene copolymer packaging materials. The migration of styrene into food depends on the characteristics of the polymer and of the food, the concentration of styrene monomer in the polymer, the diffusion coefficient, and the duration of the migration process. [41,42]. As far as the levels of xylenes (sum of *o*,*m*.*p*-xylene) and styrene in the studied cheeses are concerned, lighting and contact time promoted migration into cheeses wrapped in PE-based film (PE 200), with the exception of the Provolone sample (Figure 7). The same behaviour was observed for Taleggio and Quartirolo cheeses wrapped in PVC-based films (812 and 818). In the Provolone sample, the level of these compounds did not relate to film type or light exposure. This last factor increased the migration of xylenes and styrene to Casera cheese.

**Figure 8:**
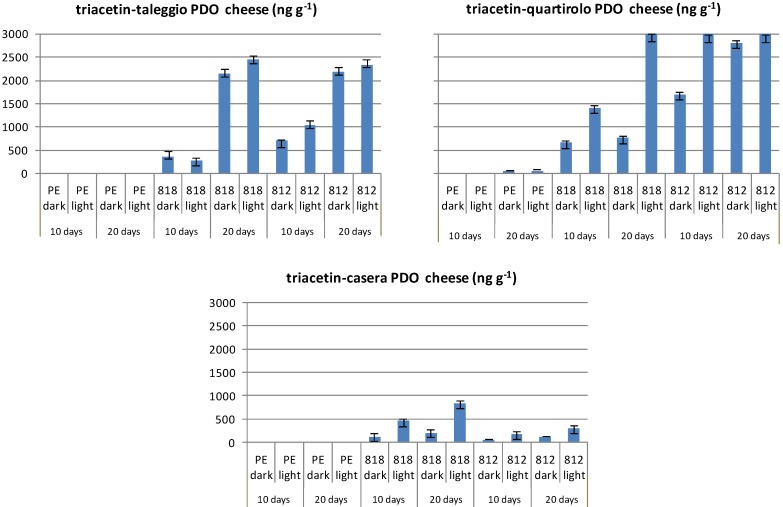
Levels of triacetin in PDO cheeses wrapped in different cling-films and stored refrigerated for 10 and 20 days in the dark or under fluorescent light (data expressed as ng·g^−1^ of cheese).

Triacetin was not present in unwrapped samples, whereas its level in wrapped samples increased as storage time augmented (Figure 8). A similar effect seemed to be caused by exposure to light. The absence of this molecule in Provolone cannot be easily explained since it largely migrated into the other cheeses studied according to storage time.

Overall, the data obtained cannot clearly discriminate or hierarchically order the different effects of light, storage time and cheese features on VOCs migration. For instance, despite fat content could favour VOCs transfer from cling-film to cheese, this chemical feature did not clearly promoted VOCs this phenomenon even though most of the VOCs previously discussed are lipophilic substances. Probably, several other factors may influence VOCs migration including cheese structure which can change according to manufacturing procedures and ripening. For instance, the acid curd of Quartirolo makes the cheese surface coarser than that of Provolone. Indeed, the hot moulding and stretching of the curd determine a smooth surface in Provolone. In the same manner, Taleggio curd softens upon ripening, whereas that of Casera does not. These structural features as well as their changes during storage following proteolysis are not easily verifiable, but they could modify the adhesiveness of cling film to the cheese surface and hence affect the overall migration of VOCs from films.

## 3. Experimental Section

### 3.1. Chemicals

All used standards were of analytical grade (purity > 99%) and were purchased from Sigma-Aldrich (Bellefonte, PA, USA). Each batch was carefully checked for possible interfering compounds as indicate in the reference method for quantitative determination of 2-EH provided by the Community Reference Laboratory for Food Contact Materials (http://ihcp.jrc.ec.europa.eu/) [31].

### 3.2. Cling-Films Samples

Three cling-films for retail use were supplied by a primary manufacturer. Two samples (812 and 818) were PVC co-polymer films of 0.012 and 0.018 mm thickness, respectively. The chemical identity of the films was verified by recording their transmittance spectra (data not shown). The 818 film was intended only for wrapping cheese with rind. The third film (PE 200) was a PE-based sample of 0.012 mm thickness. The three films were analyzed 2 months after manufacturing and hence within the declared shelf life (12 months). All the films analyzed were supplied as rolls packaged in cardboard boxes with an internal cardboard part (mandrino). Before VOC determination, the intermediate coils of each film roll were cut with a steel knife and 1 g of finely cut material was used for HS-SPME coupled to GC/MS analysis.

### 3.3. Cheeses

Four Italian Protected Designation of Origin (PDO) cheeses (Taleggio, Quartirolo, Provolone and Casera) were considered in this study. Briefly, Taleggio is a smear-coated cheese, whereas Quartirolo is a close relation of Taleggio with the differences between the two cheeses mainly due to the skimming of the milk and the lower curd pH value of the former. Provolone is a semi-hard pasta filata cheese and Casera is a semi-hard and semi-cooked cheese produced with partially skimmed milk. Whole wheels of Taleggio, Quartirolo, Provolone and Casera, were purchased from local farms. The cheeses contained 27.5%, 28.2%, 26.9% and 33.8% fat, respectively, (Table S3, Supplementary Material) (IDF-International Dairy Federation, 1993) [43]. Other cheese features are also reported in Table S3. Wheels were cut in slices with shape and weight (180–200 g) similar to the typical portioning conditions used in retail. No plastic had been used as packaging material in contact with cheeses. Pieces of cheese were then wrapped with a double layer of cling-film (812, 818 and PE200) for retail use adopting a procedure simulating actual use in retail shops. Wrapped pieces were stored in an open refrigerated (6 ± 2 °C) display cabinet either in the dark or under fluorescent light provided by a “cool white” 58 W fluorescent tube (Osram Spa, Milan, Italy). Three portions of each wrapped cheese were analysed after 10 and 20 days storage. After the exposure period, slices were cut from contact surface to a depth of 1 cm of three pieces of each cheese. The resulting individual weights and surface area in contact with film of each slice were recorded. The slices were mixed, grated and homogenized in a mortar to obtain a representative batch. The corresponding slices of unwrapped cheeses were used as blank sample. Grated cheese was used for SPME and GC/MS analysis.

### 3.4. HS-SPME and GC/MS Analysis of VOCs

Five SPME fibers and different extraction times were tested to select the optimal conditions: polydimethylsiloxane (PDMS, 100 μm), carboxen-polydimethylsiloxane (CAR-PDMS, 75 μm), polydimethylsiloxane-divinylbenzene (PDMS-DVB, 65 μm), (divinylbenzene-carbovax-poly-dimethylsiloxane (DVB-CAR-PDMS, 50/30 μm), polyacrylate (PA, 85 μm) (Supelco, Bellefonte, PA, USA). Prior to analysis, each fiber was preconditioned in the injector port of the gas-chromatograph during desorption at the temperature and for time suggested by the manufacturer. In order to test the fibers, 1 g of cling film was placed in a hermetically sealed glass vial and the samples were subjected to different extraction times (15, 45, 60, 90, 120, 180, 240 min). Finally, 5 g of cheese and 1 g of each cling-film or “mandrino” cardboard prepared as previously described underwent SPME-GC/MS analysis. All samples of cheese or film were put in a 20 mL glass vial, fitted with cap equipped with silicon/PTFE septa (Supelco), and by adding 1 mL of the internal standard solution (IS) in water (camphor, 1 µg mL^−1^, CAS 21368-68-3). At the end of the sample equilibration period (1 h), a conditioned (1.5 h at 280 °C) 50/30 µm Divinylbenzene/Carboxen™/polydimethylsiloxane (CAR/PDMS/DVB) StableFlex™ fiber (Supelco) was exposed to the headspace of the sample for extraction (3 h) by CombiPAL system injector autosampler (CTC analytics, Zwingen, Switzerland). The extraction temperature was 50 °C to obtain VOC adsorption. To keep the temperature constant during analysis the vials were maintained on a heater plate (CTC Analytics).

HS-SPME analysis was performed using a Trace GC Ultra (Thermo-Fisher Scientific; Waltham, MA, USA) Gas Chromatograph coupled to a quadrupole Mass Spectrometer Trace DSQII (Thermo-Fisher Scientific) and equipped with an Rtx-Wax column (30 m; 0.25 mm i.d.; 0.25 μm film thickness, Restek, Bellefonte, PA, USA). The oven temperature program was: from 35 °C, hold 8 min, to 60 °C at 4 °C/min, then from 60 °C to 160 °C at 6 °C/min and finally from 160 °C to 200 °C at 20 °C /min. Carry over and peaks arising from the SPME fiber were checked by regularly running blank samples. After each analysis the fibers were immediately thermally desorbed in the GC injector for 5 min at 250 °C to prevent contamination. The injections were performed in splitless mode (5 min). The carrier gas was helium at the constant flow of 1 mL^−1^. An *n*-Alkanes mixture (C_8_–C_22_, Sigma-Aldrich, St. Louis, MO, USA) was also run under the same chromatographic conditions as the samples to verify Kovats retention indices (KI) of the detected compounds confirming the identity of compounds. The transfer line to the mass spectrometer was maintained at 230 °C, and the ion source temperature was set at 250 °C. The mass selective detector parameter were set as follow: electronic impact at 70 eV, a multiplier voltage of 1456 V, and the mass spectra were obtained by collecting the data at rate of 1 scan s^−1^ over the *m/z* range of 45–450. Selected Ion Monitoring (SIM) was selected for acquisition of compounds investigated in the process of migration from cling-film to cheeses. For each compound, two ions were chosen as qualifier ions and one as quantifier ion (Table S5, Supplementary Material). Authentic compounds were analysed under the same conditions to identify and compare the retention times of peaks when available, or by comparing the Kovats retention indices with literature data. The identification of MS fragmentation patterns was performed either by comparison with those of standard compounds or using the National Institute of Standards and Technology (NIST) MS spectral database. Quantitative analyses of selected VOCs (Table S6, Supplementary Material) migrated from cling-films to cheese samples were carried out by using the internal standard procedure and expressed ng·g^−1^ using the calibration curves. All VOCs extracted from cling-films and from mandrino are expressed as µ IS equivalents g^−1^ cling-film. All analyses were performed in triplicate. Data are then presented as mean values and standard deviations.

### 3.5. Validation Parameters and Quality Control for VOCs Determination from PDO Wrapped Cheeses

All samples were prepared by weighing exactly 5 g of unwrapped cheese (obtained from representative pool of investigated PDO cheeses) in a 20 mL glass vial, and by adding 1 mL of the IS solution in water (camphor, 1 µg mL^−1^). Standard solutions of selected VOCs (1 mL), containing increasing amounts of analytes (from 0.05 µg to 15 µg mL^−1^ each) and 1 mL of IS solution in water were added to a cheese samples to obtain the calibration curves. The SPME method was also evaluated for its precision, linearity, recovery, limit of detection and quantification to evaluate the validation parameters. LOD was calculated using the equation LOD = 3.3 SD/b, where b is the slope of the calibration curve and SD is the residual standard deviation. The limit of quantification was calculated as LOQ = 3 LOD. Precision of the method was expressed as the relative standard deviation (R.D.S.) obtained from the determination of six SPME extracts (each prepared separately from a representative pool of unwrapped cheese samples). Recovery of the selected analytes was carried out at fortification levels of 50.0 ng g^−1^ by adding the known quantities of VOCs standard solution to 5 g of unwrapped cheese samples [17,18].

## 4. Conclusions

The molecules present in the volatile fraction of cling-film used in retail (based on PVC or PE) are only partially the same. The reasons are to be found in improved properties required for PVC based film used in retail, especially in terms of machinability, which prompted producers to provide film with a different composition, in terms of additives and “cling agents”. Some VOCs isolated from PVC cling-films, and hence in the cheeses wrapped in, can likely be considered as NIAS. The PE cling-film showed to possess a simpler VOC profile lacking some molecules peculiar to PVC films. The same conclusions can be drawn for cheeses wrapped in the PE cling-film. Other VOCs found in wrapped cheeses were likely to have been released either by direct transfer from the materials used for the manufacture of cling-films or from contamination of the films. This last hypothesis is certainly true for some terpenes released from all the studied cling-films, especially from the internal part of the roll. Overall, HS-SPME coupled to GC/MS proves to be a rapid and solvent free technique to screen the VOCs profile of cling-films, and to detect VOCs migration from cling-films to cheese under real retail storage conditions.

## Supplementary Materials

Supplementary materials can be accessed at: http://www.mdpi.com/1420-3049/19/7/8707/s1.
