# Supplementary material for: Determination of Volatile Organic Compounds (VOCs) from Wrapping Films and Wrapped PDO Italian Cheeses by Using HS-SPME and GC/MS
Source: Molecules. 2014 Jun 25;19(7):8707–24. doi: 10.3390/molecules19078707 (PMC6271448; doi:10.3390/molecules19078707)
Supplement: Supplementary file 1 [file molecules-19-08707-s001.pdf]

Supplementary Information

Table S1. Linearity (determination coefficient (r^2), LOD, LOQ, Precision (R.D.S) and Recovery parameters of the proposed SPME method for a 50 ng g^{-1} spike.

Investigated VOCs	LOD (ng g^{-1})	LOQ (ng g^{-1})	Recovery % (R.S.D.)	Determination Coefficient (r^2)
2-Ethylhexanol	0.99	2.97	95 (4.5)	0.9970
Ethylbenzene	1.26	3.78	90 (4.6)	0.9988
p-Xylene	1.17	3.51	85 (3.4)	0.9998
m-Xylene	1.26	3.77	82 (3.7)	0.9989
o-Xylene	0.91	2.73	87 (5.2)	0.9978
Styrene	0.95	2.84	93 (4.3)	0.9984
Triacetin	1.22	3.66	95 (4.8)	0.9978

Table S2. Global migration values of cling-film for retail use (Reg. UE 10/2011).

Cling-film	Composition-Material	Global migration (mg/dm^2 ± Standard Deviation)
PE200	PE based film	7.36 ± 1.45
812	PVC based film	8.56 ± 1.30
818	PVC based film	9.27 ± 1.20

Table S3. General features, fat, a_w, protein content and pH values of the Italian PDO cheeses.

	Taleggio	Quartirolo	Provolone	Casera
Presence of cheese rind	Yes	Yes	Yes	Yes
Thickness of cheese rind(mm)	1 mm	1 mm	2 mm	2 mm
Ripening time (months)	1	1	3	3
Shape/weight whole cheese	Rectangular 2 kg	Rectangular 1.8 kg	Round 12 kg	Round 7 kg
Protein (%)	19.7	19.9	23.8	27.1
Fat (%)	27.5	28.2	26.9	33.8
Fat (%)–dry weight	52.2	51.1	47.9	49.4
a_w	0.961	0.975	0.967	0.945
pH	5.27	4.5	5.19	5.1

Table S4. Selected reaction monitoring (SIM) parameters of the studied compounds migrated from cling-films to cheeses.

Compound	CAS Number	Qualifier Ions *m/z*	Quantifier Ion *m/z*
2-Ethylhexanol	[104-76-7]	41, 57, 112	57
Ethylbenzene	[100-41-4]	77, 91, 106	91
p-Xylene	[106-42-3]	77, 91, 106	91
m-Xylene	[108-38-3]	77, 91, 106	91
o-Xylene	[95-47-6]	77, 91, 106	91
Styrene	[100-42-5]	51, 78, 104	104
Triacetin	[102-76-1]	43, 103, 145	43

Table S6. VOCs profile by HS-SPME-GC/MS from "mandrino" of cling-films roll for retail use.

Rt [a]	Volatile Compounds [b]	Mandrino of Cling-Films for Retail Use [c]		
		**812 PVC Based**	**818 PVC Based**	**PE 200PE Based**
	Hydrocarbons			
1.63	Heptane	0.12	nd	nd
5.17	Decane	nd	0.24	1.34
16.18	Styrene	0.44	1.25	0.88
**total**		**0.56**	**1.49**	**2.22**
	Esters			
2.77	Acetic acid ethyl ester	0.56	0.77	0.23
	Terpenes			
5.49	α-Pinene	27.50	4.36	155.20
12.73	β-Myrcene	150.44	123.12	145.51
13.85	D-Limonene	1122.23	1235.13	1221.01
22.57	Citronellol	12.44	90.76	84.98
**total**		**1312.61**	**1453.36**	**1606.50**

[a] Retention time; [b] Volatile compounds: mass spectra tentatively identified using NIST 05 and Wiley 275 libraries; [c] Amount of volatile compounds expressed as μg IS equivalents g^{-1} of mandrino samples; nd = not detected.
